# Indications for Dental Specialists for Treating Obstructive Sleep Apnea with Mandibular Advancement Devices: A Narrative Review

**DOI:** 10.1155/2024/1007237

**Published:** 2024-03-31

**Authors:** Antonino Lo Giudice, Salvatore La Rosa, Vincenzo Ronsivalle, Gaetano Isola, Marco Cicciù, Giulio Alessandri-Bonetti, Rosalia Leonardi

**Affiliations:** ^1^Department of General Surgery and Medical-Surgical Specialties, Section of Orthodontics, Policlinico Universitario “Gaspare Rodolico—San Marco”, University of Catania, Via Santa Sofia 78, Catania 95123, Italy; ^2^Department of General Surgery and Medical-Surgical Specialties, Section of Oral Surgery, Policlinico Universitario “Gaspare Rodolico—San Marco”, University of Catania, Via Santa Sofia 78, Catania 95123, Italy; ^3^Department of General Surgery and Medical-Surgical Specialties, Section of Periodontology, Policlinico Universitario “Gaspare Rodolico—San Marco”, University of Catania, Via Santa Sofia 78, Catania 95123, Italy; ^4^Department of Biomedical and Neuromotor Sciences (DIBINEM), Section of Orthodontics, University of Bologna, Bologna, Italy

## Abstract

Obstructive sleep apnea (OSA) syndrome is characterized by repeated airway collapse during sleep. It determines cardiovascular, pulmonary, and neurocognitive consequences and is associated with several daytime and nighttime symptoms that influence the patient's quality of life. The contribution of the dental specialist in the clinical management of OSA patients entails participating in the screening process as diagnostic sentinels and providing adequate treatment using mandibular advancement devices (MADs). Since the treatment of OSA requires a multidisciplinary approach, including different medical specialists, dentists should have a comprehensive understanding of medical and dental factors that influence the strategy and effectiveness of OSA treatment with MAD. Such expertise is crucial in determining the appropriate treatment indications and helps clinicians establish a consolidated position within the multidisciplinary OSA team. In this regard, this review summarizes the evidence of the clinical indications for MAD treatment and provides the dental specialist with helpful information about medical, functional, and other relevant factors that should be considered during diagnosis, treatment plan, and follow-up stages. Information retrieved was organized and discussed, generating specific domains/queries oriented to the clinical management of OSA patients from the clinical perspective of dental specialists.

## 1. Introduction

Obstructive sleep apnea (OSA) is a sleep-breathing disease affecting the adult population between 9% and 38% [1, 2]. OSA is defined as the occurrence of five or more episodes of upper airway obstruction, complete (apnea) or partial (hypopnea), per hour during sleep, caused by collapsibility of the upper airway [3, 4]. The polysomnographic examination is the gold-standard diagnostic tool for analyzing respiratory disturbance, distinguishing obstructive apnea or hypopnea episodes primarily from those of central origin [5, 6]. The polysomnographic parameters used to perform diagnosis and define the severity of OSA are the apnea–hypopnea index (AHI) and the respiratory disturbance index (RDI), the latter including the number of respiratory effort–related arousals per hour of sleep in addition to apnea and hypopnea events [7, 8]. OSA is scored as mild (>5 events/hr AHI/RDI), moderate (>15 events/hr AHI/RDI), and severe (>30 events/hr AHI/RDI). OSA is associated with metabolic and cardiovascular dysfunction [9, 10], and it is considered to be a systemic life-threatening disease as patients with untreated sleep apnea have an increased risk of mortality [11, 12].

Continue positive airway pressure (CPAP) is the most effective treatment for patients with OSA [7, 13]. Despite its proven effectiveness, patients often report low adherence to the treatment [7, 14–17]. For this reason, new therapies and strategies have been advocated and explored in clinical and research settings [18]. The International and European guidelines [5, 19] clearly reported that the clinical management of OSA patients must involve a multidisciplinary group of experts, including pulmonologists, neurologists, otolaryngologists, dental specialists, surgeons, and nutritionists.

The role of the dental specialist entails participating in the screening process as diagnostic sentinels and during the treatment phase when the use of mandibular advancement devices (MADs) is advocated. In this context, extensive scientific evidence has confirmed that MADs serve as a first-line treatment approach for individuals with mild OSA as well as for adults diagnosed with moderate to severe OSA who do not respond or tolerate the CPAP [20, 21]. Systematic reviews have also focused on the efficacy of MAD therapy in relation to single specific aspects such as device design [22–24], predictive factors [25], side effects [26, 27], and treatment adherence [28]. This indicates that dental specialists play an active role in the follow-up and long-term monitoring of the patient's respiratory performance, particularly when MAD treatment is conducted in conjunction with other therapies. It also implies that dental specialists should acquire expertise in the management of OSA patients, encompassing a comprehensive understanding of medical and dental factors that influence the strategy and effectiveness of OSA treatment with MAD. Such expertise is crucial in determining the appropriate treatment indications and establishing a consolidated position within the multidisciplinary OSA team. However, the existing literature is lacking of a broad overview addressing the actual evidence-based medical and dental indications for MAD treatment and which factors the dental specialists should consider for the treatment of OSA patients; this information, collected together, would facilitate dental specialists in interacting with the other specialists of the OSA multidisciplinary team. In this regard, the present review aims to provide a summary of evidence of the clinical indications for MAD treatment and to provide the dental specialist with useful information about medical, functional, and oro-dental relevant factors that should be considered during diagnosis, treatment plan, and follow-up stages.

## 2. Materials and Methods

Database searches were performed until July 2023 to evaluate the published literature on the topic. A plentiful search strategy was applied for each database using combinations with “generic” terms (i.e., “osa” AND “mad mandibular advancement device”), and the selected databases were PubMed (https://pubmed.ncbi.nlm.nih.gov) and Scopus (https://www.scopus.com/search/form.uri?display=basic#basic). Clinical longitudinal studies dealing with adult subjects affected by OSA and in treatment with MAD as exclusive treatment or in combination with other therapies were included. The reference list of all included sources of evidence was screened for additional studies. Two authors (A.L.G. and S.L.R.) evaluated titles and abstracts retrieved from the databases, and any disagreement will be resolved after consulting another author (R.L.). No restrictions regarding the year or language of publication were applied.

## 3. Results and Discussion

**Figure 1:**
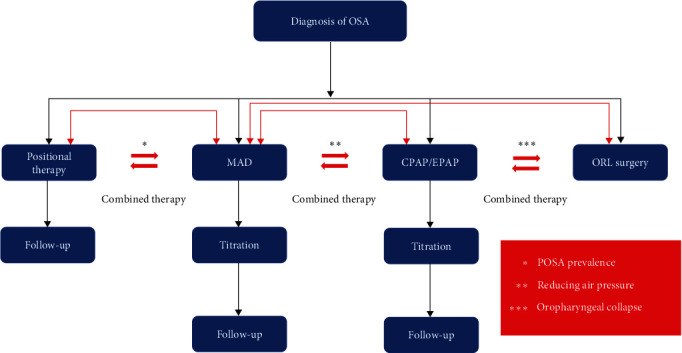
Flow-diagram showing the main treatment options for subjects affected by OSA.

**Figure 2:**
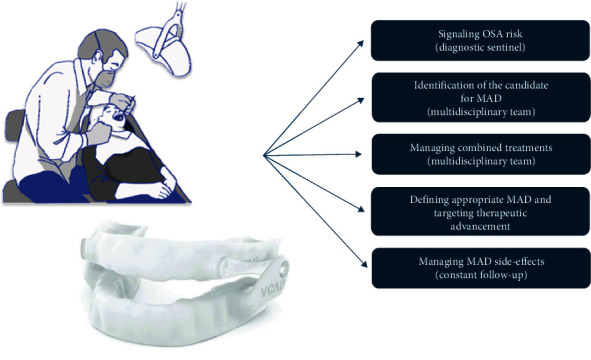
Flow-diagram showing the key rules of the dental specialist in the clinical management of OSA patients.

After duplicate removal, 2,670 records were screened by the reviewers, and a total of 209 studies were considered for final inclusion. The review included 61 clinical studies, while 30 were systematic reviews and 7 were reviews.  Table 1 presents the main characteristics of the studies included for final inclusion in the manuscript. To better address clinical indications and, considering the different types of information retrieved and the significant number of studies included, results were organized and discussed, generating specific domains/queries that covered all the information retrieved in the included studies. A schematic simplified overview of the information reported in the present review is illustrated via flow-diagram in Figures 1 and 2.

### 3.1. Factors, Not Assessable by the Dental Specialist, Predicting the Effectiveness of MAD Therapy

#### 3.1.1. Age

Younger adults have greater responsiveness to the MAD treatment compared to older subjects [29, 30]. In this regard, aging could negatively influence the effectiveness of the treatment due to the deterioration of the structure and function of the superior airway. With aging, the hypoxic response during sleep decreases, the breathing muscles have less tensile strength and fatigue resistance [31, 32], the ventilatory system during sleep is more unstable [33], the strength of the upper airway dilatator muscle reduces, and the pharyngeal closing pressure and upper airway resistance and laxity increase [34]. One study [34] found a progressive long-term (10 years) reduction of the efficacy of the treatment after the initial reduction of AHI values at 1 year and 2 years follow-up. Although the AHI values recorded at 10 years were within the clinically acceptable range of treatment success (>50% AHI reduction), this finding would encourage the necessity to monitor treatment progress, changes in lifestyle, and health status in the long term to evaluate the integration or the substitution with CPAP device.

Furthermore, compliance with MAD treatment seems to decrease with time, particularly from the 6th decade, when the patient's ability or willingness to adapt to an intraoral appliance was associated with an increased number of dropouts [35, 36]. As a consequence, the efficacy of MAD could be influenced by patients' age and dentists should dedicate time (clinical appointments) and appropriate tools (including questionnaires) for motivating older subjects to the treatment at the baseline. However, treatment motivation should not be a major concern for those subjects who have experienced CPAP since studies are almost unanimous in confirming that these patients show good adherence to the treatment with MAD.

#### 3.1.2. Gender

Gender has been claimed as a predictive factor for treatment with MAD, with female subjects being more responsive to the treatment with MADs compared to males [37]. The assumption is that the upper airway generates less resistance in females due to a smaller upper airway volume; also, female hormones may exert a protective effect on sleep-disordered breathing syndromes, which would reduce after menopause [38]. In this regard, two studies [29, 30] did not find an association between gender and treatment response, while one study [39] found that gender was a favorable predictive factor. However, since these studies did not discuss or argue such findings in detail, future studies guaranteeing the same treatment conditions between males and females are warmly encouraged to evaluate if gender may be considered a predictive factor for MAD treatment.

#### 3.1.3. Body Weight

Positive responsiveness to MAD treatment was reported in association with lower baseline values of body mass index (BMI) and neck circumference [40, 41]. Obese subjects feature an enlargement of upper airway adipose tissue, which reduces the capability of opening the retropalatal airway space during MAD wearing [42–44]. However, good responders were also found among subjects with higher BMIs and outside the currently recommended limits for clinical characteristics; thus, physiological characteristics alone should not be considered predictors of treatment responsiveness [25]. However, from the clinical perspective, a strict diet regimen represents part of the treatment, and cooperation with the nutritionist is essential to monitor treatment progress.

#### 3.1.4. Diagnostic Instrumental Parameters

Although the last European Respiratory Society guideline orientates clinicians to the usage of MAD as an alternative to CPAP in patients with mild to moderate OSA, the authors of the present review did not find agreement in the included studies in discriminating responders and nonresponders according to the AHI (or RDI) values. In this regard, lower baseline AHI values were positively associated with the responsiveness to the MAD treatment in some studies [45–48]. In contrast, other studies [29, 30, 49] did not find differences according to the severity of the AHI. However, the night-to-night variability of AHI, the different criteria used to define responsiveness and the usage of different cutoff values could be the reasons ascribable to these uncertain findings. Thus, as far as the actual evidence is concerned, AHI and RDI alone should not represent reliable parameters in predicting the treatment success with MAD, but they could be considered along with other clinical characteristics [25, 39].

Several studies reported that MAD appliances effectively reduce the AHI recorded in the supine position. Also, in three studies, MADs were considered equal to protective positional therapy in managing patients affected by positional obstructive sleep apnea (POSA) [50–52]. In this regard, the sleeping posture during PSG is an important element to discriminate the positional component within the overall AHI values [31, 53, 54], and supine-dependent sleep apneas could be a better predictor of treatment success than the disease severity measured by the total AHI [55, 56]. The positive impact of MAD appliances in reducing supine-AHI could be explained considering that the mandibular advancement mechanism is more effective in preventing the anteroposterior collapse, which, in POSA subjects, reflects a normal pharyngeal morphology with a normal airway in the lateral dimension [57, 58]. Instead, patients with apneas in the lateral position reflect a narrow airway in the lateral dimension, and problems in maintaining airway patency in the lateral sleeping position indicate a high risk of apneas [59].

#### 3.1.5. Anatomical Factors

Anatomical characteristics represent a critical etiological factor in developing OSA, considering that the reduced dimension of the bony structures and the increased expansion of the soft tissue components augment the soft tissue pressure on the airway, increasing the airway collapsibility [48, 60, 61].

Since MADs maintain airway patency by displacing the mandible and attached soft tissues forward, the assumption is that the site of collapse during sleep is an important parameter to predict the responsiveness of the treatment [62]. In this regard, primary oropharyngeal collapse, both at the level of the soft palate and tongue base, was associated with positive treatment response and should be considered a predictor of MAD response [29, 39, 63]. Also, using drug-induced sleep endoscopy (DISE) [64, 65], a positive phenotype was associated with the collapse of the tongue base, while negative phenotypes included subjects with complete concentric collapse at the level of the palate and complete laterolateral oropharyngeal collapse. This finding would also explain the positive responsiveness to the MAD treatment in POSA subjects where the anteroposterior collapse pattern plays a positive predictive role compared to the later-lateral or concentric collapse pattern.

#### 3.1.6. Cephalometric Parameters

Cephalometric analysis could represent another important tool to evaluate the predictability of the treatment with MAD at the baseline. Subjects affected by obstructive sleep apnea syndrome (OSAS) can feature an increased distance between the hyoid bone, which reduces with the usage of MAD [48, 66]. This explains the appliance's favorable biomechanics since positioning the mandible forward pulls the muscles attached to the hyoid bone, reducing the descent of the hyoid and increasing the pharyngeal airway patency. Mandibular retrognathia was found to be a common cephalometric data associated with good responders [67, 68]. One study reported that the responsiveness to the MAD treatment is not associated with skeletal parameters but rather with a larger tongue volume for a given oral cavity size, suggesting that MAD helps to correct anatomic imbalance [69].

In conclusion, the positive prediction of treatment with MAD relies not on identifying a single factor but rather on identifying a clinical phenotype featuring multiple integrated characteristics. Younger in age, lower BMI and neck circumference, the tendency to lower AHI values with the prevalent positional component, and primary oropharyngeal closure with anteroposterior collapse are baseline characteristics that would delineate a good responder phenotype. Urgent randomized clinical trials are needed to assess the predictability of both clinical, anatomical, hormonal, and instrumental parameters for the responsiveness to the treatment with MAD and to generate a predictive model or phenotype, identifying which variable is the most effective in this term.

### 3.2. Indications for Administering MAD with Other Treatments (Combined Therapy)

#### 3.2.1. MAD in Association with Positive Air Pressure Appliances

Since different treatments exhibit different mechanisms of action and the collapse of the upper airway occurs at multiple levels in most subjects with OSA [70, 71], combined treatments could improve the overall therapeutic effectiveness. In this regard, MAD may be successfully combined with CPAP and nasal expiratory positive airway pressure (EPAP) with a further reduction of the AHI compared to the single therapy [72–75]. Although nasal CPAP is the standard treatment for OSA, the high pressure generated to maintain airway patency is a common complaint that negatively influences adherence to the treatment. Since MAD primarily widens the velopharyngeal segment of the upper airway (from the hard palate to the tip of the uvula), combined treatment could reduce the air pressure required to maintain the AHI values within the normal range [76]. MAD could also act synergically with EPAP in reducing OSAS severity since MAD increases airway patency at the level of the velopharyngeal segment. In contrast, EPAP increases the hypopharyngeal segment's volume (from the epiglottis's tip to the vocal cords) [77]. Furthermore, in those patients compliant with CPAP, the alternative use of MAD is that it represents an optimal solution while traveling instead of avoiding treatment in these circumstances.

#### 3.2.2. MAD in Combination with Positional Therapy

MAD treatment and positional therapy can be complementary in the clinical management of OSA patients, particularly in those subjects with the predominant positional component [50, 52]. MAD and positional therapy do not have the same effects in expanding the pharyngeal cavity. When used together, it is possible to combine the advantages of both treatments, increasing the patency of the airway. In this regard, positional therapy increases the side lying time and decreases the proportion of supine time while MAD prevents collapse of the pharyngeal airway in the supine position to further reduce overall AHI; also, MAD cannot completely counteract the effects of gravity in the supine position. Such differences suggest that the combined therapy could lead to a higher therapeutic efficacy in patients with POSA when compared to one of the treatment modalities alone. At the same time, when patients are treated with partial success with MAD therapy, the presence of POSA should be checked, and combined therapy could be suggested in eligible patients.

### 3.3. Indication for Appliance Design and for Targeting the Therapeutic Mandibular Advancement

#### 3.3.1. Appliance Design

Several appliance designs are available in the market, with the primary distinction being between mono-bloc and dual-bloc devices. Both types of MAD were found to reduce sleepiness and daily symptoms equally [78–82]. However, it is still being determined if one design is superior to the other in improving the AHI and other parameters of respiratory disturbance. The discriminating factor between both types of appliance design is the block of mouth opening during sleep, which reduces the amount of mandibular advancement set with the bite registration. In this regard, mono-bloc appliances firmly fix the position of the mandible to the maxilla. This may explain why some studies reported greater effectiveness of mono-bloc devices compared to dual-bloc devices appliances [83, 84]. However, using anterior elastics with dual-bloc appliances can counteract the tendency to open the mouth during sleep with a similar effect to mono-bloc devices [85, 86]. It is still unclear if different designs and configurations of acrylic, such as the different sites for titration screw/tool (incisor region or premolar–molar region, upper splint or lower splint in dual-bloc devices), can influence the efficacy, the comfort, and the adherence to the treatment. However, dual-bloc devices with anterior protrusion units have the advantage of holding the mandible firmly in a protrusive position, avoiding backward mandibular rotation, and not requiring anterior elastics [35, 87, 88]. Furthermore, the vertical dimension of the MAD is an important characteristic to consider since it can negatively influence the mandibular sagittal projection (clockwise rotation). No studies reported specific indications in this regard; however, appliances tested generally featured 4–5 mm of disclosure, which is the minimum thickness necessary to produce splints or mono-blocs with adequate resistance to bite forces.

Another distinction is between customized and low-cost prefabricated heat-moulded appliances. Customized appliances would be more effective, rejecting the assumption that prefabricated appliances can be used as low-cost tools to predict the effectiveness of customized MAD treatment [89–91]. Presumably, the absence of adequate teeth coverage and lack of retention would lead patients to discontinue the treatment when using heat-molded appliances. Also, the mandibular advancement with heat-molded appliances is not reproducible since it has to be determined and controlled during the fitting procedure, generating a high standard deviation of mean mandibular advancement in the included studies and influencing the data outcomes.

#### 3.3.2. Targeted Mandibular Advancement

Targeting optimal mandibular advancement is undoubtedly one of the main clinical concerns for dentists in managing OSA patients since it remains doubtful whether there is a dose-dependent relationship between the degree of advancement and treatment outcome with MADs [92–95]. Studies assessing MAD effectiveness generally reported that 70%–80% of maximum mandibular protrusion significantly improved or normalized PSG parameters (AHI, ODI, and RDI) in at least 50% of the study population. Those studies comparing the treatment effectiveness according to different protrusion positions agreed that 75% was the most effective position, although the difference with the intermediate position (50%) was minimal and may represent the cutoff for treating mild OSAS (50% protrusion) and moderate OSAS (75% protrusion) [96–98].

Studies generally rely on subjective titration wherein the degree of mandibular advancement is progressively increased over several weeks (5–40 weeks) until an improvement or a resolution of symptoms occurs or until the patient cannot tolerate any further advancement. Studies have also used objective guided titration, which means selecting the mandibular position that has determined the abolishment of respiratory events or nonphysiologic parameters during the diagnostic instruments/procedures (DISE, PSG, oximetry) [99, 100]. Objective-guided titration is more reproducible and could avoid the overextension of mandibular protrusion, which may occur with subjective titration, and that can generate discomfort and consequently less adherence to the treatment. However, discomfort could also occur at the start of the treatment when the appliance is designed with an objectively targeted mandibular position due to the lack of the possibility for gradual adaptation. From the clinical standpoint, the presumed gold-standard titration approach may entail an objective determination of the therapeutic mandibular advancement, achievable via a personalized titration protocol. A preference for titrable devices over fixed protrusion or mono-bloc appliances is implied in this context. To ensure optimal therapy adherence, a compromise between an effective protrusive position and patient tolerance needs to be found. It is important to target the protrusive position individually in terms of tolerability (see further query) versus efficacy.

### 3.4. Indications for Managing Short-Term and Long-Term Side Effects Related to MAD Therapy

#### 3.4.1. Temporomandibular Disorders

The side effects reported by patients in treatment with MAD are represented by mild occlusal problems, discomfort, tooth sensitivity, and pain/tenderness referred to the temporomandibular joint (TMJ) or the masseter muscle area. In general, such symptoms were transient and mitigated after the first months of usage and rarely determine treatment interruption [63, 87]. Discomfort and pain-related temporomandibular disorders (TMD) are usually diffused on awakening and tend to improve during the day. Documented intra-capsular derangements are anterior displacement of the disc [101] and joint sound (clicking or crepitation) [102]. TMD-related symptoms could either increase or decrease with treatment, suggesting that MAD treatment should not be considered an absolute risk factor for the development or worsening of TMD. The contrasting findings can be associated with the different etiology of pain-related TMD and intraarticular joint disorders [7]. Subjects suffering from intraarticular joint disorders could benefit from MAD since the appliance acts as an anterior repositioning appliance. Instead, the same appliance can exacerbate symptoms in subjects with pain-related TMD, which reduces with time. In this regard, slow titration should be preferred to favoring the adaptation of masticatory structures to the advancement mandibular position. For the same reasons, dual-bloc titrable appliances may be preferred over fixed appliances, or, at least, it may be preferred during the period of acclimatization as a preliminary appliance to define the final mandibular position. These findings also suggest the importance of a preliminary TMD evaluation before administering MAD and the long-term monitoring for TMD symptoms and occlusal changes (overjet-overbite) that may require intermediate orthodontic treatment.

#### 3.4.2. Dento-Sketelal Changes

MAD can determine dento-skeletal changes equivalent to those recorded with functional appliances, although in adults [103, 104]. Such changes were generally observed through a follow-up period of 2–4 years [105, 106]. During treatment with MADs, the muscles, and the other soft tissues are stretched and try to pull the mandible back, thus transmitting a lingually directed force to the upper incisors and increasing their palatal inclination [107]. Moreover, the mandible attempts to return to its baseline position, thus transmitting a labially directed force against the mandibular incisors and increasing their vestibular inclination [108]. Skeletal changes could also occur with a reduction of intermaxillary sagittal discrepancy (ANB^). Both conditions determine a reduction of the overjet and overbite in the long term [105]. Although the clinical relevance of these findings remains questionable in most of the included studies, this review reinforces the need for OSAS patients to receive proper information about the potential occurrence of these dentoskeletal changes [109, 110]. Occlusal monitoring should also be recommended, including the necessity for adjustments of prosthetic rehabilitations to maintain proper occlusal contact and function [111].

#### 3.4.3. Oro-Facial Contraindications to the Usage of MAD

There are specific absolute contraindications to the usage of MAD. In this regard, a deeper evaluation of the oral health status is critical to evaluate and eventually manage potential side effects of the treatment [112]. Adequate dentition is fundamental for the support and retention of the MAD. Insufficient retention compromises the effectiveness of the appliance and can result in discomfort and irritation of the soft tissues, leading to reduced compliance with the treatment. While there is no specific threshold for the number of missing teeth that definitively contraindicates the use of MAD, some studies suggest that having fewer than 8–10 teeth may represent an absolute contraindication, reporting these values as exclusion criteria. However, when assessing the retention of the appliance, clinicians should also take into account the specific location of the missing teeth and the adaptability of the appliance design for edentulous conditions before excluding the option of MAD treatment. For instance, if teeth are absent in the lateral-posterior area while both incisors and molars remain intact, there may be sufficient retention in comparison to an equivalent number of missing teeth concentrated solely in the anterior or posterior region. It is essential to note that individuals with compromised periodontal health are not suitable candidates for MAD due to the potential risk of aggravating the condition, leading to tooth loss and instability of the appliance.

Limited maximum protrusion is another contraindication for administering MAD in OSA patients due to the intrinsic limitation of increasing the patency of the airway [113]. In most of the retrieved studies, protrusion values <5–7 mm are reported as exclusion criteria. An accurate clinical examination is critical to verify if the limited protrusion is related to physiological skeletal or muscular anatomical characteristics or if it is associated with TMD and can improve after specific treatment.

As a consequence, patients should undergo oral and functional clinical examination before deciding to perform instrumental analysis for targeting mandibular advancement such as nocturnal polysomnography with MAD and sleep endoscopy and, in case of irreversible contraindications, inform the other sleep medicine specialists for appropriate treatment considerations.

#### 3.4.4. General Considerations

The American Academy of Sleep Medicine (AASM) and the European Respiratory Society [5] recommended MAD as first-line treatment in mild and moderate OSA in patients without severe cardiovascular comorbidity and in severe OSA when CPAP treatment fails or is refused. As a result, the dental specialist is called to play a central role within the multidisciplinary OSA team, from the diagnosis and treatment plan to the treatment stage and throughout the follow-up period.

Despite the number of dentists qualified as specialists by the Dental Sleep Medicine Scientific Societies are increasing [114, 115], many clinicians are still reluctant in undertaking the necessary training for treating OSA patients. Suboptimal communication between sleep physicians and dentists and cotreatment management of OSA patients seem to be the main reasons for the inability of dental sleep medicine to integrate fully with the delivery of sleep medicine [114]. In this regard, the role of dentists is subordinate to sleep physicians, pulmonologists, neurologists, and otolaryngologists (due to the lack of or limited competencies in sleep disorders and respiratory functionality). The absence of close communication and a common patient-centered field could explain why clinicians are still reluctant to “go off the beaten track” of their routine clinical practice and take responsibility for the clinical management of patients suffering from OSA. The information retrieved from the included studies and collected in the form of specific clinically oriented subdomains may be helpful for dental specialists in the clinical management of OSA patients and in cooperation with the other specialists of the sleep medicine team.

## 4. Conclusions

According to the findings retrieved from the included studies, the present narrative review would suggest that:

1. The prediction of positive treatment with MAD seems to rely not on identifying a single factor but rather on identifying a clinical phenotype featuring multiple integrated characteristics.
2. In eligible patients, MAD could be used as a complementary solution with other treatment approaches (CPAP, positional therapy) to reduce apnea events within the safety range.
3. Targeted mandibular advancement, objectively recorded (i.e., via PSG or DISE) and achievable via a personalized titration protocol, could be the gold-standard titration protocol for MAD. In this context, a preference for titrable devices over fixed protrusion or mono-bloc appliances is implied.
4. Side effects of MAD, which were represented by mild occlusal problems, discomforts, tooth sensitivity, and pain/tenderness, referred to the TMJ or the masseter muscle area. Dento-skeletal changes can be considered equivalent to those recorded with the usage of functional appliances, although in adults.
5. An accurate clinical examination is critical to exclude oral baseline contro-indications to the usage of MAD.

## Figures and Tables

**Table 1:** Characteristics of the included primary studies.

Author/year/study type/country	Sample	Age	Type of appliance	OSA severity a T0	Measurments	Period	Results
Aarab, 2010, RCT, Netherlands	20 patients	49.5 ± 8.1	MAD, set at a constant vertical dimension with 0%, 25%, 50%, and 75% of the maximum protrusion	21.6 ± 11.1 AHI	PSG, ESS	39 weeks	The AHI values in the 50% and 75% positions were significantly lower than those in the 25% position

Aarab, 2011, RCT, Netherlands ^*∗*^	64 patients: 21 MAD, 22 CPAP, 21 placebo	50.4 ± 8.9	MAD and CPAP	21.4 ± 11.0 AHI	PSG	18 months	Discomfort in wearing, tenderness in the masseter muscle region upon awakening, and feeling of a changed occlusion upon awakening

Aarab, 2011, RCT, Netherlands	57 patients: MAD (*n* = 20) nCPAP (*n* = 18) Placebo (*n* = 19)	50.3 ± 9.1	MAD and nCPAP	22.1 ± 10.8 AHI	PSG	6 months	MAD is effective in the supine position

Almeida, 2013, clinical trial, Canada	22 patients	53.8 ± 12.1	MAD and CPAP	30.7 ± 23.1 AHI	PSG, ESS, SAQLI	3 months	A combination of therapies (MAD and CPAP) allows greater flexibility of treatment and opportunity for ongoing adherence in circumstances where CPAP cannot be used

Barnes, 2004, RCT, Australia	104 patients	47.0 ± 0.9	MAD, CPAP, and placebo	21.3 ± 1.3 AHI	PSG, neurobehavioral testing, 24-hr ambulatory blood pressure, and echocardiography, maintenance of wakefulness test, Stanford Sleepiness Scale, ESS	11 months	77% of subjects achieved at least 70% of the maximum possible protrusion. With this degree of protrusion, 56.1% subjects achieved a reduction in the AHI of at least five events per hour

Benoist, 2017, RCT, Netherlands	99 patients: 51 MAD, 48 SPT	49.2 ± 10.2	MAD and SPT	11.7 (9.0–16.2) AHI	PSG, ESS, FOSQ	3 months	The SPT and MAD were equally effective in reducing the AHI and ODI in POSA patients
							For patients using SPT, the AHI will likely decrease in all sleeping positions when MAD is added

Berg, 2020, RCT, Norway	104 patients: 55 CPAP, 49 MAD	49.6 (9.0)	MAD and CPAP	16.3 (12.4–23.0) AHI	SF36, PSQI, PSG	12 months	Seven (14.3%) in the MAD treatment group had quit treatment, all reporting not being compliant to treatment

Bishop, 2014, RCT, USA	24 patients	47.4 ± 2.6	Klearway and TAP3	19.3 ± 4.6 AHI	ESS, SAQLI, RSR	3 months	Age negatively influences a patient's ability or willingness to adapt to an intraoral appliance
							Neither appliance proved to be more effective than the other in any AHI classification for any variable recorded

Blanco, 2005, RCT, Spain	24 patients: 12 advanced, 12 control	55.6 ± 11.8 advanced, 53.0 ± 12.7 control	Two models of MAD with 5 mm were applied, one model with an advance of 75% and one without	33.8 (14.7) AHI advanced, 24.0 (12.2) AHI control	PSG, ESS, SF-36, FOSQ	3 months	Patients in the advanced group presented a decrease in the number of apneas in the supine position, suggesting that the device could be particularly effective in cases of position-dependent OSA
							The group treated with the MAD, which advances the mandible, presented a greater reduction, and more than half of the patients in this group achieved complete control of OSA symptoms

Bloch, 2000, RCT, Switzerland	24 patients	50.6 ± 1.5	Monoblock, Herbst	26.7 ± 3.3 AHI	ESS, PSG	5 months	The AHI during treatment with the OSA-Herbst device but not with the OSA-Monobloc device was significantly correlated with the baseline AHI
							Both IOAs improved sleep-disordered breathing and measured snoring. These effects were even more pronounced for the OSA-Monobloc than for the OSA-Herbst device, but the differences were not significant
							Seven patients complained of temporomandibular joint pain, four of muscle discomfort, and three of dental discomfort. The prevalences of these side effects were identical for the OSA-Herbst and OSA-Monobloc appliances

Brown, 2021, CT, Australia	105 patients	45 ± 12	MAD	30 ± 19 AHI	PSG, MRI	3 months	Participants without PMR tendon had greater mandibular advancement, greater anteroposterior airway diameter increase, and increased odds of complete response in those who tolerated treatment
							About 7% of participants overall were unable to acclimatize to MAS because of ongoing pain, jaw locking, or other and withdrew, the majority of these being PMR tendon absent

Campbell, 2009, RCT, New Zeland	28 patients: 12 objective advancement, 16 subjective	49.8 ± 12.6 objective, 48.1 ± 10.6 subjective	MAD	26.5 ± 12.0 AHI objective, 25.4 ± 7.4 AHI subjective	PSG, ESS, questionnaires	6 weeks	There were no significant differences in BMI, neck circumference, and baseline AHI between the “success” subjects with a complete response to treatment and the poor responders in the treatment failure category
							Neither titration method (self-titration or fixed at 70% protrusion) was significantly superior

Chan, 2010, CT, Australia	69 patients	50.5 ± 10.1	MAD	27.0 ± 14.7 AHI	PSG, MRI	8 weeks	There were no significant differences between responders and nonresponders with respect to age, gender, or BMI

Chan, 2010, CT, Australia	35 patients	53.7 ± 11.9 responders, 55.8 ± 10.1 nonresponders	MAD	29.3 ± 15.7AHI responders, 24.1 ± 11.2 AHI nonresponders	PSG, nasopharyngoscopy	6–8 weeks	There were no significant differences between responders and nonresponders with respect to age, sex, body mass index, or baseline AHI. An increase in the velopharyngeal cross-sectional area with mandibular advancement was significantly associated with a treatment response on polysomnography

Chen, 2008, CT, China	70 patients	50.0 ± 9.6	MAD	0.0–68.0 RDI	Dental model analysis system	7 years 4 months	The study provides evidence of significant occlusal changes, but none of the 70 patients stopped OA treatment because of this

Chen, 2019, CT, Netherlands	64 patients	58 (48.5, 67) responders, 59.0 (48.3, 64.8) nonresponders	MAD	22.2 (15.5, 30.4) AHI responders, 26.9 (14.3, 39.6) AHI nonresponders	CBCT, PSG	6 weeks	Body mass index (BMI) was significantly smaller at baseline in the responders than in the nonresponders. Neck circumference (NC) of the OSA patients was also significantly smaller at baseline in the responders than in the nonresponders

Dal-Fabbro, 2014, RCT, Brazil	39 patients	47.0 ± 8.9	Placebo, MAD, CPAP.	42.3 ± 4.5 AHI	PSG, ESS	6 months	Supine and nonsupine AH events both improved with CPAP and MAD, with the first one which had a stronger effect

De Almeida, 2002, CT, Brazil ^*∗*^	7 patients	47.4	MAD	13.20 AHI	PSG, MRI	9 months	One patient had an anterior displacement with reduction, and two patients had anterior displacement without reduction. In the other two patients, osteophytes were seen in both joints

De Britto-Teixeira, 2013, RCT, Brazil	19 patients	48.6 (9.6)	Placebo and Twin Block	16.3 ± 7.2 AHI	PSG	10 months	The use of TB produced a reduction in AHI from 16.3 (SD = 7.2) to 11.7 (SD = 9.4). The use of WRAP (placebo) yielded an increase in AHI from 16.3 (SD = 7.2) to 19.6 (SD = 14.8)

De Corso, 2015, CT, Italia	65 patients	44.26	MAD	21.4 ± 6 AHI	DISE, ESS, Berlin	3 months	The presence of an anteroposterior pattern of closure and absence of the latero-lateral one at the level of the palate, as documented during pretreatment DISE, are associated with therapeutic success in mild/moderate OSA patients treated with custom-made MADs

De Ruiter, 2018, RCT, Netherlands	99 patients	49.2 ± 10.2	MAD and SPT	11.7 (9.0–16.2) AHI	PSG, position sensor, FOSQ-30, ESS	12 months	Supine AHI decreased to a similar extent in the two groups
							The most common adverse events in both groups were persistent snoring and persistent tiredness. Tooth pain, temporomandibular dysfunction, and open bite

Deane, 2009, RCT, Australia	27 patients	49.4 ± 11.0	MAD and TSD	27.0 ± 17.2 AHI	PSG, questionnaries, ESS	12 weeks	Analysis of the effect of the appliances on AHI in supine and other body positions during sleep demonstrated that AHI between baseline and MAD was significantly different

Dieltjens, 2015, RCT, Belgium	20 patients	52.5 ± 10.5	MAD and SPT	24.6 ± 10.2 AHI	PSG, questionnaires	6 months	MAD therapy was effective in reducing both supine AHI and non-supine AHI when compared to baseline
							A combination of SPT + MAD therapy further reduces the sleep apnea severity when compared to the individual treatment modalities

Doff, 2010, RCT, Netherlands	103 patients: 51 MAD, 52 CPAP.	49 ± 10	MAD and CPAP	39 ± 31 AHI	PSG, lateral cephalogram	2 years	Mainly dental changes in the craniofacial morphology in the MAD group compared with the CPAP group following 2 years of treatment

Doff, 2012, CT, Netherlands	103 patients: 51 MAD, 52 CPAP.	49 ± 10	MAD and CPAP	39 ± 31 AHI	PSG, dental models in articulator	2 years	A decrease in overjet, overbite, number of occlusal contact points, and a different anterior–posterior relationship are dental changes most likely to occur

Doff, 2012, CT, Netherlands	103 patients: 51 MAD, 52 CPAP.	49 ± 10	MAD and CPAP	39 ± 31 AHI	Mandibular function and impairment questionnaire (MFIQ), function impairment rating scale (FIRS), questionary, PSG	2 years	The occurrence of (pain-related) TMDs increases in the initial period of MAD therapy but tends to return to baseline values during a 2-year follow-up

Doff, 2013, RCT, Netherlands	103 patients: 51 MAD, 52 CPAP	49 ± 10	MAD and CPAP	39 ± 31 AHI	PSG, ESS, FOSQ, SF-36	2 years	Older, obese, and with predominantly severe OSAS patients switched from MAD therapy to CPAP therapy
							Tooth pain, temporomandibular joint pain, myofascial pain, dry mouth, and excessive salivation. Long-term oral appliance therapy and CPAP may result in dental changes in patients with OSAS

Edwards, 2016, RCT, USA	14 patients	51.8 ± 2.3	MAD	29.6 ± 5.3 AHI	Two PSG (clinical and research), with and without OA	1 week	Baseline anatomy/collapsibility (i.e., Vpassive) and loop gain were independent predictors of patients likely to gain the greatest benefit from MAD therapy. Trend for responders to have more severe OSA without their devi

El-Sohl, 2011, CT, USA	10 patients	56.9 ± 6.1	Auto-CPAP, MAD	23.5 ± 13.4 AHI	PSG, ESS	3 days	The combination therapy was successful in reducing optimal CPAP pressure and normalizing AHI in selected patients with OSA
							Two patients noted a feeling of pressure in the face, and one patient complained of early morning, non-persisting discomfort in the mouth and temporomandibular joint

Engleman, 2002, RCT, United Kingdom	51 patients	46 ± 9	MAD and CPAP	31 ± 26 AHI	Questionnaires, home sleep monitoring	4 months	CPAP preference with higher body mass index

Fleury, 2004, CT, France	40 patients.	57 ± 9	MAD	46 ± 21 AHI	PSG, questionnaires, ESS, VAS	18 months	At the first assessment, which was performed at 80% of MMA, only four patients presented sufficient clinical and oximetric improvement to allow polysomnography. These four patients were in the success group. For the remaining 36 patients, advancement was continued beyond the initial advancement. 25% of advancements were motivated by abnormal ODI, despite the resolution of the symptoms
							For all of the patients in groups (dental class of Angle) 2 and 3, mandibular advancement had to be stopped due to temporomandibular discomfort

Fransson 2002, CT, Sweden	65 patients	54.8 ± 9.0	MAD	14.0 ODI	Cephalogram, PSG	2 years	The SNB angle decreased significantly, because of posterior rotation of the mandible and a significant increase in anterior face height. We found lower incisors to be proclined after 2 year

Fransson, 2022, RCT, Sweden	314 patients	55 (49;65) non-POSA and 54 (47;63) POSA	Monobloc and bibloc	29 (17;39) AHI non-POSA, 23 (14;30) AHI POSA	Night at-home polygraphic study, ESS, PGIC	1 year	The subgroup of subjects with severe OSA at baseline showed the greatest improvements in AHI. The decrease in supine AHI was significantly greater among subjects with POSA, whereas the decrease in nonsupine AHI was significantly greater in the non-POSA group
							The original efficacy studies showed equivalent efficacy of the 2 types of appliances, so they were analyzed together in this study. Advance the mandible to 75% of maximal capacity or by at least 5 mm

Friedman, 2010, CT, USA	87 patients	45.70 ± 11.47	Two nontitratable one-piece MAD and a titratable two-piece device	39.96 ± 23.70 AHI	ESS, PSG, VAS	2 months	Patients in the study with prior surgery did not fare better than those naïve to surgery

Garcia-Campos, 2016, CT, Mexico	30 patients	49.7 ± 12.45	MAD	22.45 ± 6.14/hr AHI	ESS, questionnaires, PSG	3 months	The most commonly found side effect was excessive drooling, which lasted for about a month and then disappeared with no treatment. The second most frequent side effect was dental pain, which was also self-limited. Four patients reported no side effects

Gauthier, 2009, RCT, Canada	23 patients	47.9 ± 1.6	Two MADs	9.4 ± 1.1 RDI	PSG, FSS, ESS, FOSQ, questionnaires	2 years	The RDI was also reduced by both MAA with no difference between appliances in the supine position or in non-REM sleep
							Both appliances had 4 mm advancement, but the silencer was statistically more efficient at reducing the RDI

Geoghegan, 2015, RCT, China	45 patients	52	Bibloc and monobloc MADs	21.1 (14.2–50.1) AHI	Lateral cephalogram, PSG, ESS	26 weeks	Both MADs resulted in similar significant cephalometric changes around the hyoid bone position and soft palate length
							After treatment, there was a highly significant reduction in AHI with the monoblock and with the twin block. The monoblock demonstrated a significantly better result than the twin block
							Changes were seen in several other measurements (SNB, mandibular plane angle, overjet, overbite, and face height) with both MADs

Ghazal, 2009, RCT, Germany	103 patients: 51 IST, 52 TAP	55.5 ± 10.6	Two MADs	32 ± 6 AHI IST, 37 ± 8 AHI TAP	PSG, ESS, PSQI, questionnaires	2 years	51% of the patients with the IST and 79.2% with the TAP demonstrated complete treatment success

Gogou, 2022, CT, Greece	50 patients: 34 DISE, 16 control	48.8 ± 12,3	MAD	31.7 ± 17.3 AHI	DISE, PSG, questionnaires	8 weeks	An increase of upper-airway dimensions during mandibular advancement during DISE may have predictive value regarding the likelihood of successful treatment with a MAD
							By using DISE, the full short-term efficacy of MAD treatment was achieved with less initial mandibular protrusion than in the control group

Gotsopoulos, 2002, RCT, Australia	73 patients	48 ± 11	MAD, control device	27.1 ± 15.3 RDI	ESS, questionnaire, PSG	10 weeks	The MAD produced a 52% reduction in mean RDI and a significantly higher mean minimum arterial oxygen saturation (MinSao_2_) compared with the control device
							A significantly higher proportion of patients experienced side effects with the MAD than with the control device, namely, jaw discomfort, tooth tenderness, and excessive salivation

Huang, 2023, RCT, China	60 POSA patients: 20 SPT, 20 MAD, 20 combined	39.20 ± 10.92 SPT, 41.55 ± 11.79 MAD, 40.75 ± 10.51 SOT	SPT, MAD	19.21 (11.77–23.90) AHI SPT, 18.58 (16.10–24.55) AHI MAD, 14.85 (11.93–26.59) AHI SOT	PSG, ESS, QSQ	6 months	After 3 and 12 months of follow-up, MAD and SPT have comparable effects on improvements in the AHI and compliance in subjects with mild-to-moderate POSA
							Treatment with both SPT plus MAD can combine the advantages of the two methods and achieve complementary results

Isacsson 2019, RCT, Sweden	302 patients: 146 bibloc, 156 monobloc	54 (12.2) bibloc, 55 (11.4) monobloc	Bibloc and monobloc MADs	27 (14.2) AHI bibloc, 25 (14.1) AHI monobloc	PSG, ESS, FOSQ	2 months	The effect of reducing AHI was significantly equivalent between the two appliances

Ishiyama, 2017, RCT, Japan	25 patients. 13 jaw exercises, 12 placebo	51.4 ± 9.7	MAD	21.5 ± 10.0 AHI	PSG, questionnaires, ESS, PSQI, VAS	3 months	Arthralgia at the 1-month evaluation. Disc displacement developed in one subject in the JE-group at the 2-week and 1-month evaluations and in two or three subjects in the PE-group across evaluation periods. Chewing pain and jaw-opening pain in the morning at the 1-month evaluation

Jo, 2018, CT, Korea	79 patients	44.7 ± 13.1	MAD	17.3 ± 5.6 AHI	Questionnaires, DISE	2 years	This study revealed that the degree of obstruction at the levels of the velum and epiglottis were significantly decreased after long-term oral appliance therapy

Johal, 2007, CT, UK	50 patients	51.1 (7.1)	MAD	17.3 (5–30) AHI	ESS, PSG, EMG	8 weeks	A highly significant increase in the EMG activity was observed in two upper airway dilatory muscles and a muscle of mastication, following the placement of MAS in awake OSA patients

Johal, 2017, RCT, United Kingdom	25 patients	44.9 (SD 11.5)	Ready-made and custom-made MADs	13.3 (10.9–25) AHI	Visi-Lab Greyflash at home, ESS, FOSQ, SF-36, OAOQ	7 months	The MRDc resulted in a statistically significant difference in terms of total treatment success (96%; *n* = 24). The MRDr resulted in a total treatment success of 64% (*n* = 16)

Johnston, 2002, RCT, Northern Ireland	21 patients	55.10 ± 6.87	MAD, placebo	31.93 ± 21.18 AHI MAD; 30.69 ± 18.82 AHI placebo	ESS, questionnaires, Edentrace II	12 weeks	MAD was significantly more effective than the placebo in improving the outcome measures
							The most commonly reported complication was excessive salivation when wearing the appliance. Some subjects reported temporary occlusal changes in the morning. Temporary TMJ discomfort on waking was common

Kato, 2000, RCT, Japan	37 patients	49.0 (27.1–66.6)	Three MADs with 2-, 4-, and 6-mm mandibular advancements	26.0 (11.2 to 72.0) ODI	Endoscopy, oximetry	1 week	Obese patients with severe nocturnal desaturation may not be appropriate candidates for MAD therapy. The presence of severe OP and hypopharyngeal narrowing may be an alternative explanation for the poor responses to the MADs
							Step-advancement of mandibular position resulted in a dose-dependent reduction of closing pressure of the passive pharynx, (2) successful improvement of nocturnal oxygenation appeared to be achieved when the MAD reduced the closing pressure of the passive pharynx below atmospheric pressure, and (3) each 2-mm mandibular advancement coincided with approximate 20% improvement of the number and severity of nocturnal desaturations
							Excessive salivation and transient discomfort or pain of the temporomandibular joint for a brief time after awakening were commonly reported

Kazemeini, 2022, RCT, Belgium	10 patients	48.0; 41.5; 55.6	MAD with subjective, objective PSG titration and DISE titration	21.3; 17.5; 26.8 AHI	PSG, DISE	4 months	Comparable amounts of titration and corresponding efficacy in terms of AHI reduction and reduction in subjective symptoms were found among the three titration methods. In titrationSubj, the relief of subjective complaints may lead to premature interruption of the titration and a suboptimal treatment outcome. On the other hand, objective titration may induce discomfort at the start of MAD treatment, therefore possibly making habituation more difficult

La Mantia, 2018, RCT, Italy	40 patients	49.6 ± 11.6 AB, 47.5 ± 10.2 BA	Bibloc and monobloc MADs	28.5 ± 5.7 AHI	PSG, ESS, SAQLI	22 weeks	Use of the monoblock MAD should be considered when patients with OSAS choose MAD treatment, as it was more efficient in improving objective OSAS parameters compared to twinblock MAD

Lai, 2019, CT, Australia	22 patients	49 ± 12	MAD and EPAP	15 (10,34) AHI	PSG	6 weeks	The addition of oral and oral plus nasal EPAP valves to a novel MAS device resulted in stepwise reductions in OSA severity

Lai, 2022, CT, China	105 patients: 65 with retrognathia (33 CPAP, 32 MAD), 40 no (20 CPAP, 20 MAD)	46.72 + 10.19 with, 46.78 + 11.37 without	MAD and CPAP	37–38 mean AHI	PSG, cephalometry, questionnaires	12 months	Mandibular advancement device treatment of severe OSA patients with mandibular retrognathia is superior to that of severe OSA without mandibular retrognathia in terms of AHI and ODI

Lawton, 2005, RCT, UK	16 patients	44.8 (range 24.0–68.4)	Twin block, Herbst	45.5 (29.0–68.0) AHI	Questionnaires, domiciliar sleep study, ESS, SF-36, VAS	14 weeks	There was no significant difference in the median AHI scores produced by treatment with the Herbst (24.5, *n* = 16) and TB (34.0, *n* = 15) appliances
							With Herbs appliance, muscular discomfort was experienced by 56% initially, but this improved to 25% after 4 6 weeks. With the TB, there was a reduction from 50% to 19%. Initial TMJ discomfort improved from 69% to 31% and 38%–19%, respectively, for the Herbst and TB appliances. An abnormal bite was experienced initially by 69% of Herbst and 38% of TB, and they reduced to 56 and 21. Dry mouth from 63 to 56 with Herbst and 75–63 with TB. Excessive salivation 31–19 for Herbst and 44–31 for TB

Ma, 2020, CT, China	42 patients	41.5 ± 9.0	MAD	23.4 ± 11.5 AHI	Rhinospirometry, rhinomanometry, magnetic resonance imaging, home sleep testing, and PSG baseline	1 year	It was found that the dose-dependent relationship between AHI reduction and mandibular protrusion was nonlinear, and the overall success and normalization rate entered a relative plateau stage after approximately 70% MMP

Makihara, 2022, RCT, Japan	32 patients: 17 50%, 15 75%	62.2 ± 1.90	MAD 50% and 75% of maximum mandibular protrusion	22.3 ± 13.49 AHI	ESS, PSG	4 months	Effective treatment across both mandibular advancement groups was more often documented in females compared to males
							While treatment success rates were higher with 50% mandibular advancement compared to 75% mandibular advancement, this difference was not statistically significant in patients with mild to moderate OSA

Marco-Pitarch, 2018, CT, Spain	41 patients	54.5 ± 10.3	MAD	22.5 ± 16.8 AHI	ESS, PSG, VAS	6 months	The higher the SaO_2_ Min. initial value, the smaller the improvement produced by MAD, and the larger the arousal index initial value, the larger the improvement after placement of the oral appliance. Only gender and Fujita index were statistically significant

Marklund, 1998, CT, Sweden	26 patients: 12 Posa, 14 non-POSA	59 POSA, 54 non-POSA	MAD	41 (range, 16–70) AHI	PSG	2 months	This study demonstrates that supine-dependent sleep apnea is a strong predictor of successful treatment with the MAD in patients with obstructive sleep apnea

Marklund, 2004, CT, Sweden	619 patients: 160 snoring, 459 OSA	51 men (range, 25–74). 55 women (range, 30–75)	MAD	16 (range, 0.0–76) AHI	PSG	2 years	Women with sleep apnea were more likely than men with sleep apnea to have treatment success with the mandibular advancement device. Supine-dependent sleep apneas, mild disease, and an increase in mandibular advancement predicted treatment success among the men, while mild sleep apnea was associated with treatment success in the women
							Discomfort, including excessive salivation or a feeling of awkwardness when wearing the device, was the main cause of the poor tolerability of the device. Insufficient effects on snoring or odontologic problems, i.e., symptoms from the craniomandibular system, periodontal disease, or changes in occlusion during treatment, were other explanations for a failure to accept the device

Marklund, 2015, RCT, Sweden	91 patients: 45 MAD, 46 placebo	49.8 (10.6) MAD, 54.1 (9.4) Placebo	MAD	15.6 (9.8) AHI MAD, 15.3 (10.5) AHI placebo	ESS, KSS, OSLER, SF-36, FOSQ, PSG	4 months	It was observed that patients using a MAD slept more in the supine position than in nonsupine positions, indicating that the effect of an oral appliance in reducing sleep apneas was even more effective than the results of the AHI revealed
							The AHI was 6.7 (SD, 4.9) for the MAD group, which was significantly lower than in patients using the placebo device (16.7 (SD, 10.0)); Snoring appeared less than once a week during treatment with the oral appliance, which was less than with the placebo device
							Jaw pain, tooth pain, hypersalivation, and bite changes. Adverse effects were more common with MAD than with the placebo device

Marklund, 2016, RCT, Sweden	9 patients	68.1 (60.0–76.3)	MAD	17.3 (IQR 9.7–26.5) AHI	PSG, ESS	16.5 years	Both the overjet and the overbite decreased significantly during treatment with OA. Deteriorations in OSA severity and a loss of OA efficacy were found in the present small sample of patients treated continuously for more than 15 years with this method

Mehta, 2001, RCT, Australia	28 patients	48 ± 6.9	MAD, control oral plate	27 ± 17 AHI	PSG, questionnaires, ESS, cephalometric radiographs	4 weeks	AHI with the MAD is positively correlated with neck circumference and baseline AHI and negatively correlated with the width of the retropalatal airway and angulation of the mandibular plane to the anterior cranial base

Mosca, 2022, CT, Canada	58 patients	51.6 ± 8.0 (28–70)	MAD	31.4 ± 23.0 (10.0–105.3) AHI	Home sleep test, AI, and heuristic prediction method	3 nights/6 months	Irritation to teeth, jaw, or gums; dryness; excessive salivation; and sleep disturbance as a result of OAT. The percentage of participants who reported experiencing bite changes as a result of OAT was 27.3

Mostafiz, 2011, CT, Australia ^*∗*^	53 patients	49.5 ± 11.8	MAD	33.014.4 AHI	Lateral cephalogram	2 months	Treatment nonresponders were significantly older with more severe OSA than complete responders. Maxillary length and upper-facial height were significantly shorter in complete responders than in partial responders. BMI, tongue area, oral area, and tongue/oral CSA ratio were considered as independent variables for predicting %AHI using multiple linear regression

Neill, 2002, RCT, New Zeeland	19 patients	47.7 ± 10.1	Two MADs	22.2 ± 19.8 (SD) RDI	PSG, questionnaires	6 weeks	The mean mandibular protrusion in eleven subjects was 61.5% of the maximum, which was lower than ideal and may have reduced the success of this treatment. However, we found no relationship between the degree of advancement and measures of OSAS improvement

Ng, 2006, CT, Australia	12 patients	51 ± 9	MAD	22.0 ± 2.6 AHI	PSG, nose mask, nasendoscopy	8 weeks	Patients with oropharyngeal closure were significantly more likely to have complete responses with MAD therapy than were patients with velopharyngeal closure. The AHI supine reduced

Nikopolou, 2020, RCT, Netherlands	57 patients: 20 MAD patients, 18 nCPAP patients, and 19 placebo	52. 0 ± 9.6	MAD, CPAP, placebo	21.4 ± 11.0 AHI MAD; 20.1 ± 9.0 AHI CPAP; 19.5 ± 8.4 AHI placebo	MFIQ, FIRS	6 months	Low frequency of clinical signs of TMD pain in mild to severe OSA patients
							Clinical signs of temporomandibular disorders who also expressed a desire for treatment of their TMD complaints, an unhealthy periodontium (periodontal pockets >5 mm), dental pain, and/or inadequate retention possibilities for an intraoral appliance were excluded as well

Niżankowska-Jędrzejczy, 2014, CT, Poland	38 patients: 22 OSAS MAD, 16 control	52.50 ± 8.33 OSA, 54.06 ± 12.09 control	MAD	24.00 (15.70–31.25) AHI	PSG, blood samples	6 months	Supine AHI significantly decreased from 36.50 to 15 and 12 at 3 and 6-month follow-up

Op De Beeck, 2019, CT, Belgium	100 patients	47.6 ± 10.0	MAD	21.0 ± 11.2 AHI	PSG, DISE	3 months	The presence of tongue base collapse during baseline DISE examination is strongly correlated to favorable MAD response in patients with OSA. Patients with complete concentric collapse at the level of the palate (CCCp) and/or complete laterolateral oropharyngeal collapse (CLLCop) during DISE tend to deteriorate under MAD treatment
							Mild, temporary side effects, as is usual during the startup of any MAD treatment

Op De Beeck, 2021, CT, Belgium	36 patients	48.5 (45.8–51.1)	MAD	23.5 (19.7–29.8) AHI	PSG, ESS, VAS	3 months	MAD responders were slightly younger than nonresponders. MAD treatment significantly improved AHI, supine AHI, and nonsupine AHI. A greater reduction in AHI was associated with lower loop gain, a higher arousal threshold, a lower response to arousal, moderate collapsibility, and weaker muscle compensation

Pepin, 2019, RCT, France	198 patients: 100 TALI, 98 ONIRIS	51 (SD, 12)	Heat-molded and custom-made MADs	26.6 SD 10.4 AHI	ESS, VAS, SF-12, PSG	2 months	After 2 months, both treatments significantly improved AHI per hour, and scores for SF-12 (both the physical and mental subscores), Pichot fatigue and depression scales, Epworth sleepiness scale, and snoring with no significant differences between the two MADs
							The most frequently reported side effects were dental pain, temporomandibular joint pain, discomfort related to MAD volume in the mouth, muscular pain, and muscle aches. Excessive salivation and gag reflex were observed in the ONIRIS group

Perck, 2020, CT, Belgium	100 patients	47.6 ± 10.0	MAD	14.6 (9.3–24.0) AHI	Nasopharyngoscopy, PSG	3 months	The current study indicated a relationship between a prominent uvula (C-shaped palate) and a negative response to MAD treatment

Petri, 2008, RCT, Denmark	93 patients: 33 MAD, 30 MNA, 30 placebo	50 ± 11 MAD, 50 ± 10 MNA, 49 ± 10 placebo	MAD, MNA, placebo	39.1 ± 23.8 AHI MAD, 32.6 ± 22.0 AHI MNA, 34.3 ± 26.3 AHI placebo	PSG, ESS, SF-36, QOL	4 weeks	MAD had a significant effect on AHI, calculated separately for the supine and nonsupine sleeping positions
							AHI, Epworth score, and vitality in the MAD group differed significantly from that in the MNA group and no-intervention group
							Two patients could not tolerate the appliance; one patient suffered loosening of the teeth, and one suffered pain of the temporomandibular joint

Petri, 2019, CT, Denmark	62 patients	51 (range 27–65)	Custom-made, monobloc MAD	34 (range 6–117) AHI	PSG, cephalometry, acoustic reflectometry	13 weeks	POSA is indicative for success, and nonsupine AHI is inversely related to success. Cephalometry was not predictive
Pitsis, 2002, RCT, Australia ^*∗*^	23 patients	50 ± 10 (29–64)	MAD-1 and MAD-2 with 4 and 14 interincisal opening	21 ± 12 (6–47) AHI	Questionnaires, PSG, ESS	2 months	The amount of vertical opening induced by the appliance does not have an impact on treatment efficacy to any great extent
							Excessive salivation (48% versus 57%), dry mouth (26% versus 22%), tooth grinding (22% versus 13%), and gum irritation (22% versus 13%) between MAD-1 and MAD-2, there was a trend toward a greater proportion of patients reporting jaw discomfort with MAS-2 (48% versus 70%)

Quinnell, 2014, RCT, UK	90 patients	50.9 (11.6)	Thermoplastic ‘boil and bite' device, semi-bespoke device, and bespoke MAD	13.8 (6.2) AHI	PSG, ESS, FOSQ, SAQLI, SF-36, (EQ-5D-3 L)	5 months	The response was significantly associated with baseline BMI and contemporaneous BMI. Baseline AHI, ESS, gender, age, and compliance were not associated with treatment response
							Mouth problems/discomfort and excess salivation with SP2 performing best for both

Randerath, 2002, RCT, Germany	20 patients	56.5 ± 10.2	CPAP, ISAD	17.5 ± 7.7 AHI	PSG	3 months	The patients in whom effectiveness was demonstrated in the first ISAD application differed from nonresponders by their significantly younger age and heavier weight
							Two patients noted a feeling of pressure in the mouth; eight patients complained of early morning, nonpersisting discomfort in the mouth and temporomandibular joint

Remmers, 2017, CT, Canada	202 patients	48.4 (26–70) part 2, 49.8 (24–76) part 1	MAD	25.5 (10.5–65.1) ODI part 1, 31.1 (10.3–74.6) ODI part 2	In-home feedback mandibular positioner. Home PSG	Four nights	Some participants reported having tooth and/or gum discomfort during the FCMP test

Ringqvist, 2003, RCT, Sweden	67 patients: 30 MAD, 37 UPPP.	48.9 (46.3–51.4) years MAD, 51.0 (49.1–52.9) years UPPP	MAD, UPPP	17.9 (2.9) AHI dental, 19.9 (3.0) AHI UPPP	Lateral cephalometry	4 years	The vertical positions of the maxillary incisors (the distances incision superius (IS)-NSL and is-ML) and the mandibular incisors (the distance incision inferius (II)- NSL) changed significantly. The mandible rotated posteriorly (the mandibular plane angle increased by 0.5°). As a consequence of the posterior rotation of the mandible, the distances II-NSL and IS-ML increased

Rose, 2002, RCT, Germany	26 patients	56.8 ± 5.2	Two MADs	16.0 ± 4.4 RDI	PSG, VAS, portable somnograph	20 weeks	Both appliances investigated are effective in treating patients with mild OSA and can be used as an alternative treatment option. Concerning the RDI and AI, the nonretentive activator proved to be statistically more effective than the retentive Silencor® appliance
							The initial side effects of the Silencor were higher salivation and complaints of pain in the gingiva and teeth. Side effects were more frequent with the activator; in addition to increased salivation, seven patients (30%) complained of pain in the TMJ and f tenderness in the masseter muscle

Sanner, 2002, CT, Germany	15 patients	57.2 ± 8.9	MAD	19.8 ± 14.5 AHI	MRI, PSG, questionnaires	4 weeks	Dental discomfort, xerostomia, excess salivation, bite change, and temporomandibular joint pain

Sari, 2011, CT, Turkey ^*∗*^	24 patients: 12 Klearway, 12 MAD	39 ± 4.2	KW and MAD	18, 8 ± 7, 3 AHI KW, 17.9 ± 6.8 AHI MAD	PSG, ESS	1 month	Klearway and MAD appliances are both effective in the treatment of mild and moderate OSA patients. An appliance (Klearway) that provides advancement of 85% of mandibular protrusion to open the upper airway was more effective in reducing the number of high apneic events during sleep than one (MAD), which provides 75%. Mandibular advancement device (MAD) should be preferred in mild OSA patients rather than moderate OSA patients
							Mild pain in the TMJ and muscle tenderness. 25% of MAD had gum irritation (not in Kw, thanks to the thermoelastic material). 17% of KW had lower anterior tooth discomfort (due to increased retention) in the morning

Shi, 2023, RCT, Netherlands	31 patients: 16 MAD-H and 15 MAD-S	48.5 (±13.9)	MAD-H (Herbst appliance); MAD-S (SomnoDent)	16.6 (±6.7) /hr AHI	ESS, PSG, CBCT	3 months	The AHI, AHI-nonsupine (not the AHI supine), and ODI reduced significantly with MAD in situ in the total group
							Although the freedom of vertical opening is different between MAD-H and MAD-S, it seems that the respiratory outcomes were not affected by this design feature
							Sensitive teeth and painful jaw muscles were 3–4 times more frequent in the MAD-H group compared to the MAD-S group, which might be due to the different design features. Painful temporomandibular complaints. 19% of MAD-H and 13% of MAD-S had changes in occlusion in the morning

Suga, 2014, CT, Japan	20 patients: 7 rigid, 13 semi-rigid	58.1 ± 7.6 rigid, 57.9 ± 11.4 semi-rigid	Rigid and semi-rigid MAD	22.0 ± 13.8 AHI rigid, 20.5 ± 8.5AHI semi-rigid	PSG, TC	3 years	Neither the change of the occlusion nor TMDs occurred in the both groups

Sutherland, 2014, RCT, Australia	78 patients	49.3 ± 11.1	CPAP, MAD	30.0 ± 12.7/hr AHI	PSG	3 months	For MAD response by definition 1 (MAD AHI <5/hr), only baseline AHI and age were significant predictors. In predicting MAD response by definition 2 (MAD AHI <10/hr), the combination of baseline AHI, age, and CPAP pressure was significant. By definition 3 of MAD response (≥50% AHI reduction), only age and neck circumference, but not CPAP pressure, had predictive value

Sutherland, 2016, CT, Australia	35 patients	53.7 ± 11.9 responders, 55.8 ± 10.1 nonresponders	MAD	29.3 ± 15.7AHI responders, 24.1 ± 11.2 AHI nonresponders	PSG, nasopharyngoscopy	6–8 weeks	Excess upper airway soft tissue within the intramandibular space area, between gonion points and menton, is associated with a poor response to MAD treatment

Sutherland, 2018, CT, Australia	142 patients	56.3 ± 11.0	MAD	28.7 ± 17.5 AHI	PSG, nasopharyngoscopy, spirometry, craniofacial photography	4 months	Responders tended to have a longer lower face, increased facial axis angle, and reduced maxillary and mandibular position angles, suggestive of maxillary/mandibular retrusion. We did not find any sex differences in the relationship between treatment response and any of the clinical or phenotypic predictors

Sutherland, 2018, CT, Australia	80 patients	57.6 ± 11.2	MAD	26.4 ± 15.4 AHI	Nasopharyngoscopy, PSG	15 weeks	Our qualitative scoring system indicated a reduction in the level of collapse induced by the Müller maneuver with mandibular advancement. A stabilization of the airway with mandibular advancement would be expected

Svanholt, 2015, CT, Denmark	27 patients	52.6	MAD	10.6 and 111.7 (mean 39.1) AHI	Lateral cephalogram	4 weeks	BMI was significantly smaller in the success treatment group compared with the no-success treatment group. OSA patients with retrognathia of the jaws responded successfully to MAD treatment, and the retrognathia of the maxilla was found to be the most important factor for the MAD treatment outcome

Tan, 2002, RCT, UK	24 patients	50.9 ± 10.1	CPAP, MAD, MAD II	22.2 ± 9.6 AHI	PSG, questionnaires, ESS	2 months	Initial jaw discomfort early in the morning, but only one could not adapt to the device. There were no dental problems. Some degree of discomfort in the TMJ, facial musculature, or teeth on waking have been reported previously; these are normally mild and improve with time

Tegelberg, 2003, RCT, Sweden	74 patients: 38 : 50% MAD; 36 : 75% MAD	51.8 (49.0 ± 54.6) group 50–54.4 (52.4 ± 56.4) group 75	MAD	16.2 (2.9) AHI 50, 18.9 (4.7) group 75	PSG	1 year	Nine patients in group 50 withdrew before the 1-year follow-up for the following reasons: 3 could not tolerate the dental appliance. Ten patients in group 75 withdrew before the 1-year follow-up for the following reasons: 1 could not tolerate the dental appliance, and 2 had TMJ pain on movements of the mandible

Tegelberg, 2020, RCT, Sweden	302 patients: 146 bibloc, 156 monobloc	55 (11.4) bibloc, 55 (10.7) monobloc	Bibloc and monobloc MADs	25 (12.9) AHI bibloc, 23 (13.6) AHI monobloc	PSG	1 year	Although there was a greater reduction in the AHI in the bibloc group, the proportion of responders defined as having an AHI <10 at the 1-year follow-up was 68% in the bibloc group and 65% in the monobloc group
							Treatment-related adverse events were generally mild and transient and occurred in 39% and 33% of bibloc and monoblock, respectively

Tong, 2020, RCT, Australia	16 patients	48 ± 11	MAD and CPAP	26 ± 13 AHI	PSG	12 weeks	Combination therapy with CPAP and a novel MAD can normalize pharyngeal pressure swings and lower CPAP requirements by 40% compared with CPAP alone

Umemoto, 2019, CT, Japan	52 patients: 23 twin-block, 29 fixed MAS	52.9 ± 10.7 twin-block, 53.8 ± 8.6 fixed	Bibloc and monobloc MADs	20.6 ± 11.5 AHI twin-block, 21.4 ± 15.2 AHI fixed	PSG, ESS, cephalogram radiographs	3 months	Significant improvements were observed in the AHI after using either the twin-block adjustable MAS allowing mouth opening or the fixed MAS, but the proportion of responders was significantly greater in the fixed group than in the twin-block group. In addition, the fixed group exhibited a significant improvement in the snoring index, arousal index, and desaturation rate
							Patients with anodontia, severe malocclusion, severe periodontitis, or temporomandibular joint (TMJ) pain dysfunction syndrome were excluded

Uniken Venema, 2020, RCT, Netherlands	103 patients: 51 MAD, 52 CPAP.	61 ± 8 MAD, 59 ± 10 CPAP	MAD and CPAP	31.7 ± 20.6 AHI MAD, 49.2 ± 26.1 AHI CPAP	PSG, ESS, FOSQ, Short Form Health Survey (RAND-36), and a questionnaire evaluating adherence	10 years	The relapse in AHI could possibly be explained by a change in lifestyle, health status, or aging. With aging, there is an increase in pharyngeal closing pressure and upper airway resistance, due to a decrease in upper airway dilatator muscle strength

Van Den Bossche, 2022, CT, Belgium	100 patients	48.3 (10.0)	MAD	15.6 (10.4–23.5) AHI	Computational fluid dynamics, DISE, nasendoscopy, PSG, CT	3 months	Tongue base collapse during baseline is a positive predictor for successful MAD treatment for OSA. Furthermore, the presence of CCCp is an adverse DISE phenotype towards MAD treatment outcome
							It is solely the presence of a prominent uvula (C-shaped position of the soft palate) during tidal breathing that remains strongly correlated with MAD treatment deterioration after multimodal labeling

Vanderveken 2008, RCT, Belgium	35 patients	49 ± 9	Custom-made MAD, thermoplastic MAD	13 ± 11 AHI	PSG, VAS, ESS	9 months	A custom-made MAD is more efficacious than a prefabricated MAD made from thermoplastic material in the treatment of snoring and mild sleep apnea
							No serious side effects were noted with either MAD

Vecchierini, 2019, CT, France	312 patients: 77 women, 235 man	57 women, 52 men	MAD	26.5 women, 30 men AHI	PSG, questionnaires	3–6 months	The treatment success rate was higher in women than in men, particularly in severe OSA. Complete response was also more common in women versus men across a range of AHI thresholds. Smaller neck circumference at baseline as a statistically significant independent predictor of MRD success in women. Treatment response in the severe OSA group was significantly better in women versus men. Decreases in AI and AHI were only independent predictors of treatment success and complete response in men
							Women who experienced side effects were more likely to discontinue therapy than men. At least one side effect was reported by 55% of women and 49% of men. Mouth or temporomandibular joint pain was responsible for 60% of treatment discontinuations

Vroegop, 2013, CT, Belgium	200 patients	46 ± 9	Custom-made simulation bite. A custom-made, titratable, duobloc MAD	19 ± 13 AHI	PSG, DISE, ESS, VAS	3 months	The presence of palatal collapse at baseline evaluation was also associated with treatment response
							Unable to tolerate the device throughout the night, choking sensations or side effects such as tooth tenderness and dry mouth, or a combination of thereof, and claustrophobia during MAD wear
							The presence of hypopharyngeal collapse at baseline evaluation showed a tendency toward an association with a less favorable treatment outcome

Walker-Engstrom, 2002, RCT, Sweden	72 patients: 32 dental appliances, 40 UPPP	20–65	MAD	17.9 (2.9) AHI dental, 19.9 (3.0) AHI UPPP	PSG	4 years	One patient (3%) was not able to occlude his teeth in the same way as before treatment and reported TMJ pain on movement of the mandible. (1) Five patients (15%) reported unilateral TMJ sounds (four patients reported clicking, and one patient reported crepitation). Three of these patients had reported these symptoms before treatment

Walker-Engstrom, 2003, RCT, Sweden	77 patients: 40 MA 75% and 37 MA 50%	50.4 (47.7–53.1) MA 75%, 54.3 (52.2–56.4) for the 50% MA group	MAD 50% and 75%	47.0 (5.1) AHI MA 50%, 50.4 (4.7) AHI MA 75%	PSG, ESS, questionnaires	6 months	The patients who were normalized had a significantly lower mean value for BMI
							The somnographic variables (AI, AHI, ODI, and SI) decreased significantly between baseline and the 6-month follow-up in both groups. No significant difference between the two groups
							One patient (3%) in the 50% MA group was not able to occlude his teeth in the same way as before treatment. Five patients (12%) in the 75% MA group reported complaints of pain from the TMJ after an average time of 3 months (one resolved, four switched to MA 50%). Headache was significantly reduced after 6 months in the 75% MA group but not in the 50% MA group. In the 75% MA group, one patient could not tolerate the dental appliance. In the 50% MA group, five patients withdrew before the 6-month follow-up; two patients could not tolerate the dental appliance

Wang, 2015, CT, China	42 patients	47 ± 10	MAD	27 ± 19 AHI	Questionnaires, cephalometry, PSG	4 years	Skeletal changes, however, were predominantly induced by dental changes. Increases in lower and total anterior facial heights resulted from changes to the maxillary and mandibular incisors. Downward rotation of the mandible was caused by the retroclination of the maxillary incisors and the proclination of the mandibular incisors through incisal guidance, and lower and total anterior facial heights were thus increased

Wilhelmsson, 1999, RCT, Sweden	95 patients: 49 dental, 46 UPPP	49.3 (46.8–51.9) MAD, 51.0 (49.1–52.9) UPPP	MAD	18.2 (15.7–20.8) AHI MAD, 20.4 17.4–23.3 AHI UPPP	PSG, questionnaires, pharyngoscopy, home sleep	1 year	The positive effect of the dental appliance was also independent of whether the predominant obstruction determined by FPMM was in the oropharynx, the hypopharynx, or both
							Pain and tenderness from the temporomandibular joint were recorded at the 1-year follow-up

Yanamoto, 2021, RCT, Japan	15 patients	50.0 (31.5–69.0)	Semi-fixed and fixed MAD	12.5 (8.9–17.0) AHI	PSG, a portable sleep test device	10 weeks	There was no significant treatment difference in AHI, 3% ODI, and lowest SaO_2_ between the semi-fixed and fixed MAD
							The fixed MAD resulted in a significantly higher incidence of TMJ pain compared to the semi-fixed MAD

Yang, 2015, RCT, China	40 patients: 20 UPPP and 20 UPPP + MAD	46.7	MAD	55.53 ± 5.61 AHI	PSG, CT	3 years	The combination of UPPP surgery and MAD therapy can further improve upper airway ventilation on the basis of OSAHS surgery, remitting airway obstruction symptoms, significantly reducing the recurrence rate, and improving the patient quality of life

Zhou, 2012, RCT, China	16 patients	45.23 years from 26.3 to 55.4	Bibloc and monobloc MADs	26.38 ± 4.13 AHI	Questionnaires, PSG, cephalometric radiography	6 months	Both appliances manifested the potential to improve AHI, AI, and hypopnea index (HI), with a more statistically important improvement for AHI and AI in the case of the monoblock appliance

^*∗*^Articles included from review.

## Data Availability

The datasets used and/or analyzed during the current study are available from the corresponding author upon reasonable request.
